# Editorial: Alcohol Consumption and Liver Diseases: From Pathology to Phytotherapy

**DOI:** 10.3389/fphar.2022.848334

**Published:** 2022-02-16

**Authors:** Songtao Li, Zhenyuan Song, Ping Yao, Jiangjiang Qin

**Affiliations:** ^1^ School of Public Health, Zhejiang Chinese Medical University, Hangzhou, China; ^2^ Department of Kinesiology and Nutrition, University of Illinois at Chicago, Chicago, IL, United States; ^3^ School of Public Health, Tongji Medical College, Huazhong University of Science & Technology, Wuhan, China; ^4^ The Cancer Hospital of the University of Chinese Academy of Sciences (Zhejiang Cancer Hospital), Institute of Basic Medicine and Cancer (IBMC), Chinese Academy of Sciences, Hangzhou, China

**Keywords:** alcoholic liver disease, hepatic steatosis, liver injury, phytochemicals, phytotherapy

Globally, there are 2.4 billion people who consume alcohol with a high proportion of 39.5% of heavy episodic drinkers among them (GBD 2016 Alcohol Collaborators, 2018). Emerging evidence showed that harmful alcohol consumption is associated with more than 200 diseases and injuries, and leads to 3.3 million deaths worldwide annually (Seitz et al., 2018). As an important site for alcohol metabolism, the liver becomes the main target organ of chronic and heavy alcohol consumption-induced injury, which is referred to as alcoholic liver disease (ALD). The spectrum of ALD develops from steatosis to steatohepatitis, with some individuals ultimately progressing to fibrosis, cirrhosis, and even hepatocellular carcinoma. The perniciousness of ALD has not attracted enough attention, since the incidence rate of ALD in the population is much less than that of other liver diseases, such as non-alcoholic fatty liver disease. But in fact, ALD is the main source of terminal liver disease in clinic. Alcohol is responsible for about 25% of cirrhosis deaths and 30% of all liver cancer deaths annually (Asrani et al., 2021). Considering the significance of ALD on public health, Frontiers in Pharmacology organized this Research Topic entitled “Alcohol Consumption and Liver Diseases: from Pathology to Phytotherapy” to present recent advances in this field.

## Pathological Mechanism of Alcoholic Liver Disease

During the past decades, progress has been made on the understanding of pathological mechanism(s) of ALD. Ethanol oxidation by alcohol dehydrogenase and aldehyde dehydrogenase leads to a reduction of the NAD^+^/NADH ratio, and then disturbs fatty acid oxidation, glycolysis and glucogenesis, and the tricarboxylic acid cycle, contributing to hepatic steatosis; meanwhile, ethanol oxidation by the microsomal ethanol oxidizing system causes excessive reactive oxygen species generation, which further attacks biological macromolecules resulting in liver injury (Ceni et al., 2014). Emerging evidence shows that alcohol-mediated modification on intracellular biological macromolecules, such as RNA, DNA, and protein, as well as epigenetic modification, contributes to the development of ALD (Smathers et al., 2011; Chen et al., 2020; Dou et al., 2020; Dou et al., 2021; Zhang et al., 2021).

Impaired intestinal flora homeostasis, disturbance of flora metabolites, and loss of intestinal barrier integrity, as well as gut-liver crosstalk have been recognized as novel contributors to ALD (Hartmann et al., 2015). Recognition of pathogens and pathogen-associated molecules is determined by germline-encoded pattern recognition receptors, including toll-like receptors (TLRs). TLR4 activation has been reported to be implicated in the pathological process of ALD (Hritz et al., 2008), however, as another fundamental immunoreceptor, the biological function of TLR9 in ALD is still not fully illustrated. In this Research Topic, Hao et al. reported that mice with TLR9 deficiency were more susceptible to chronic alcohol-induced oxidative stress, endoplasmic reticulum (ER) stress, and liver injury; while, TLR9 activation by CpG oligodeoxynucleotides improved ethanol metabolite and acetaldehyde-induced hepatocyte injury. However, they also observed that TLR9 deficiency protected against alcohol-induced inflammation in mice liver, indicating the bidirectional regulation of TLR9 on liver damage and inflammation in ALD (Hao et al.). This study enhanced our understanding of TLR9 in ALD. Alcohol consumption is usually accompanied by food intake. Therefore, nutritional factors, such as dietary fatty acids, modulate susceptibility to ALD. Diet with high n6-polyunsaturated fatty acids (PUFAs) aggravates, while, n3-PUFAs ameliorates early steatosis in ALD (Tull et al., 2009; Patterson et al., 2012). Warner et al. established an early alcoholic hepatitis (AH) model, and reported that enhancing endogenous n3/n6-PUFAs ratio by transgenic Fat-1 protected AH in mice. This study provided the first clue that enriching the endogenous n3-PUFAs level is a potential way to improve AH.

## Phytotherapy of Alcoholic Liver Disease

Currently, there are no accepted therapies available to prevent or cure ALD. Alcohol abstinence is the most common and most effective therapy to attenuate and even reverse the disease. The potential mechanism(s) involved in alcohol abstinence-improved alcoholic fatty liver remain unclear. Pi et al. explored the molecular basis of alcohol withdrawal-regulated metabolism reprogramming. Their findings enhanced our understanding of the biological mechanisms of alcohol abstinence on ALD treatment. Besides alcohol abstinence, searching for effective compounds to treat ALD is another essential and urgent task to prevent the development of ALD or treat it clinically. Phytochemicals, possessing strong abilities in defending hepatic oxidative stress, endoplasmic reticulum stress, and inflammation, which are hallmarks of ALD, are potential candidates for ALD therapy. Here, in this Research Topic, Yan et al. summarized 23 kinds of phytochemicals with strong anti-ALD effects based on clinical studies or animal experiments. Via employing a more appropriate model to mimic heavy alcohol drinkers with a Western diet, Jiang et al. reported that cannabidiol, a botanical component extracted from marijuana, protected chronic ethanol plus high-fat high-cholesterol diet-induced hepatic steatosis, oxidative stress, and inflammation in ALD mice. Gao et al. reported for the first time that kinsenoside, an active ingredient extracted from *A. roxburghii*, possessed the robust ability to alleviate chronic alcohol feeding-induced oxidative stress, ER stress, and liver injury via stimulating AMPK-dependent autophagy. Liu et al. reported anti-hepatocellular carcinoma activity of a new saponin *Dioscorea* Zingiberensis via regulating LncRNA TCONS-00026762. Since hepatocellular carcinoma is one of most adverse outcomes in ALD, the beneficial roles of *Dioscorea* Zingiberensis on alcohol-associated liver cancer is worthy of further study. Additionally, accumulating clinical evidence confirmed the effectiveness of traditional Chinese medicine (TCM) in ALD treatment. Different from Western medicine, TCM is composed of many components and acts on multi-targets. In this Research Topic, we collected one TCM prescription, named Zhi Gan prescription, which was composed of six herbal medicines and containing 138 predicted active components. The authors reported the beneficial role of Zhi Gan prescription against hepatic steatosis and liver injury, which are the same hallmarks of ALD (Qin et al.). It is worth looking into this anti-ALD effect in a future study.

## Conclusion

In summary, this special issue contains eight representative articles, including seven original articles and one review. Evidence from these articles has greatly promoted our scientific understanding of the pathological mechanisms and biological prevention and treatment of ALD. This topic has so far attracted wide attention with over 12,700 views.
